# A Systematic Review and Meta-Analysis of the Relationship Between Hospital Volume and the Outcomes of Percutaneous Coronary Intervention

**DOI:** 10.1097/MD.0000000000002687

**Published:** 2016-02-08

**Authors:** Xiaojun Lin, Hongbing Tao, Miao Cai, Aihua Liao, Zhaohui Cheng, Haifeng Lin

**Affiliations:** From the Department of Health Administration, School of Medicine and Health Management (XL, HT, MC, ZC, HL); and Family Planning Research Institute, Center for Reproductive Medicine, Tongji Medical College, Huazhong University of Science and Technology (AL), Wuhan, China.

## Abstract

Previous reviews have suggested that hospital volume is inversely related to in-hospital mortality. However, percutaneous coronary intervention (PCI) practices have changed substantially in recent years, and whether this relationship persists remains controversial.

A systematic search was performed using PubMed, Embase, and the Cochrane Library to identify studies that describe the effect of hospital volume on the outcomes of PCI. Critical appraisals of the methodological quality and the risk of bias were conducted independently by 2 authors. Fourteen of 96 potentiality relevant articles were included in the analysis. Twelve of the articles described the relationship between hospital volume and mortality and included data regarding odds ratios (ORs); 3 studies described the relationship between hospital volume and long-term survival, and only 1 study included data regarding hazard ratios (HRs). A meta-analysis of postoperative mortality was performed using a random effects model, and the pooled effect estimate was significantly in favor of high volume providers (OR: 0.79; 95% confidence interval [CI], 0.72–0.86; *P* < 0.001). A systematic review of long-term survival was performed, and a trend toward better long-term survival in high volume hospitals was observed.

This meta-analysis only included studies published after 2006 and revealed that postoperative mortality following PCI correlates significantly and inversely with hospital volume. However, the magnitude of the effect of volume on long-term survival is difficult to assess. Additional research is necessary to confirm our findings and to elucidate the mechanism underlying the volume–outcome relationship.

## Abstract 2

Supplemental Digital Content is available in the text

## INTRODUCTION

Over the past few decades, numerous studies have investigated the relationship between procedural volume and the outcomes of percutaneous coronary intervention (PCI);^1–5^ the primary conclusion derived from these studies is that high-volume hospitals achieve better outcomes than low-volume hospitals. In recent years, however, PCI practices have changed substantially. These changes include the use of low-profile balloons, drug-eluting stents, glycoprotein IIb/IIIa inhibitors, and intra-aortic balloon pumps. Additionally, the rates of PCI have been declining steadily because of improvements in cardiovascular disease prevention and the implementation of alternative medical therapies that preclude the use of PCI,^6^ which may affect the persistence of the volume–outcome relationship.

Copious convincing evidence has demonstrated the existence of a volume–outcome relationship following PCI; however, methodological problems in many of those studies have been noted.^7–10^ For example, the data from these studies usually have a 2-level structure of patients within hospitals,^11^ but the cluster effect is ignored in many studies, which may result in an overestimation of the strength of the volume–outcome relationship.^10^ Studies using administrative data are more likely to report significant results than studies using clinical data.^7^ However, in recent years, more studies have taken the above-mentioned limitations into consideration and provided more robust estimates.

Although a previous meta-analysis combined several observational studies and described a significant relationship between hospital volume and in-hospital mortality,^12^ the study was limited because only 10 studies were available, and any articles published after 2008 were not included. Furthermore, the relationship between hospital volume and long-term outcomes following PCI, including survival, has not been reviewed previously. An improved understanding of the volume–outcome relationship may have important clinical and policy implications because centralizing PCI may improve patient outcomes. Given the above-mentioned evidence, our aim was to evaluate the strength of the relationship between hospital volume and mortality following PCI by conducting a meta-analysis and to analyze the relationship between hospital volume and survival by conducting a systematic review.

## METHODS

### Search Strategy and Selection Criteria

We performed a systematic literature search using PubMed, Embase, and the Cochrane Library using the following keywords: (percutaneous coronary intervention) AND (hospital volume OR provider volume OR institutional volume) AND (mortality OR survival rate) (see Table 1; Supplemental Content, which describe the search strategy in detail). The literature search was last conducted on May 21, 2015. Because volume is not well indexed in electronic databases, we formulated the search terms to make them as sensitive as possible to ensure that no publications were missed. Reference lists of relevant articles were hand-searched to identify additional articles. Two reviewers (Lin and Cai) independently screened both the titles and the abstracts of all retrieved articles.

To best reflect the modern PCI practices and perioperative management, we only included the articles published after 2006. Studies were selected using the following inclusion criteria:

1. the subject of the study was PCI;
2. the relationship between hospital volume and the outcomes of PCI was investigated;
3. the study used primary data (ie, letters, editorials, and reviews were excluded);
4. the study reported >1 of the predefined outcomes of interest, including postoperative mortality and survival;
5. the study reported odds ratios (ORs), hazard ratios (HRs), relative risks (RRs), or adjusted rates;
6. the results were adjusted for differences in case-mix, specifically age and gender;
7. the study did not describe the results obtained at a single hospital;
8. the article was written in English;

Following primary selection, the full-text articles were obtained and underwent additional screening using the following exclusion criteria:

1. Multiple publications based on the same database; only the most recent or most informative article was included;
2. no definition of procedural volume as a distinct number (eg, a continuous variable) or cut-off values (studies that defined volume as “specialization” were excluded);
3. no postoperative outcomes (ie, morbidity, mortality, survival, or quality of life);
4. publication before 2006.

Any discrepancies regarding either the inclusion or the exclusion of specific studies were resolved via discussion and consultation with a third investigator (Tao).

### Data Extraction and Quality Assessments

The data were extracted by 1 reviewer into structured summary tables and checked for accuracy by a second reviewer. Any disagreements were resolved via discussion until a consensus was reached. The quality and generalizability of the studies were assessed based on key domains considered fundamental for observational studies.^13^

### Data Synthesis

Hospital volume was measured as the annual number of PCI cases performed by hospital or institution. The study outcomes were mortality and survival (time to death) following PCI. Mortality was defined as either all-cause death in the hospital or death within 30 days following PCI, and survival was defined using a minimal follow-up period of 3 months.

A meta-analysis was performed to determine the relationship between hospital volume and postoperative mortality. Pooled estimated effect sizes were calculated using the adjusted outcomes of the highest volume group, as opposed to the lowest volume group (reference). If the highest volume group was used as the reference, the results were transformed (1/effect size) to fit the statistical model. Studies without a multivariate analysis and studies that did not report either ORs or RRs were excluded from the meta-analysis. A random effects model was used to account for excepted heterogeneity.^14^ Heterogeneity was quantified using the *Q*-statistic and *I*^2^ test.^15,16^ We conducted a sensitivity analysis to explore possible explanations for heterogeneity and to assess the impact of various subgroups. Publication bias was assessed using an Egger's regression intercept.^17^ A meta-regression analysis was performed to determine the cut-off values for hospital volume, the proportion of patients undergoing PCI for acute lesions, the proportion of patients treated with stents, the proportion of male patients, and the study publication year using a fixed-effects regression test. The data were analyzed using Comprehensive Meta-Analysis version 3 (Biostat, Englewood, NJ). This study was conducted according to the check lists of Meta-analysis Of Observation Studies in Epidemiology (MOOSE). All analyses were based on previous published articles; thus there was no requirement for ethical approval. All reported *P*-values are 2-sided.

## RESULTS

### Study Characteristics

**FIGURE 1:**
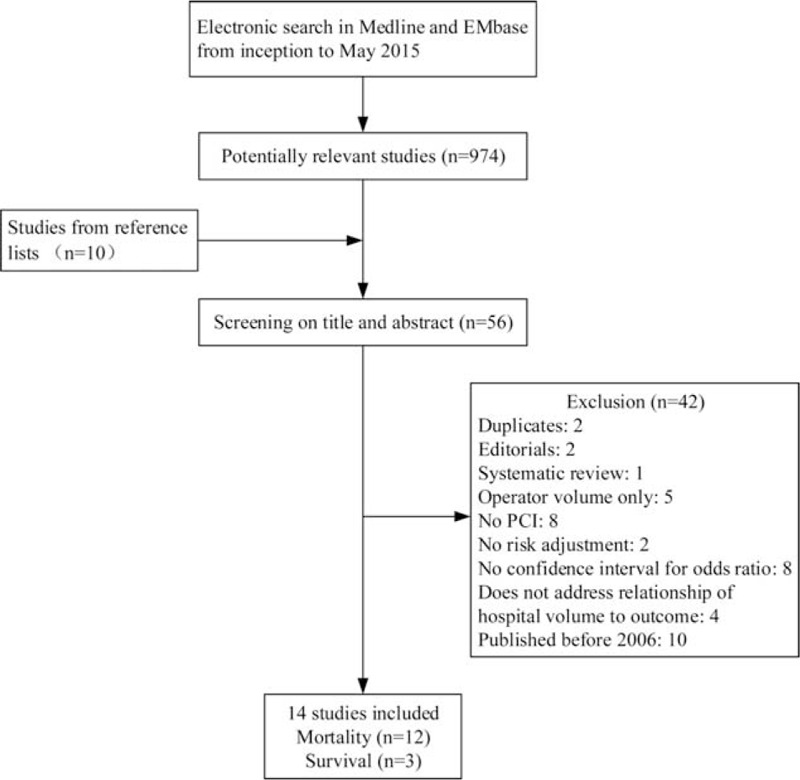
Flowchart of the literature selection process. PCI indicates percutaneous coronary intervention. PCI = percutaneous coronary intervention.

**FIGURE 2:**
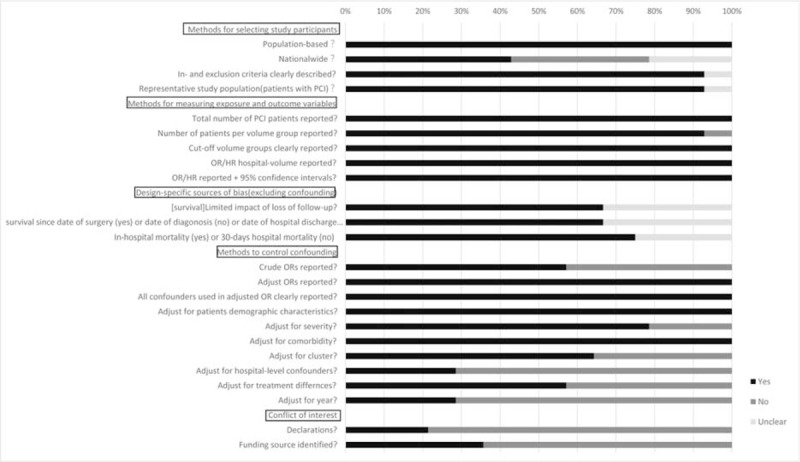
Quality assessment of all 14 included studies.

Our initial search identified 974 potentially relevant articles regarding volume–outcome relationships following PCI. After screening the title and abstracts and applying the selection criteria, 14 articles were included in our review (Figure 1). In accordance with predefined outcomes, mortalities were examined in 12 studies,^1,2,18–27^ and survival in 3 studies.^18,28,29^Table 1 presents the characteristics of the 14 studies from 6 countries included in this review.^1,2,18–29^ Six studies were from the United States, 2 were from Europe, and 4 from Asia. Although we excluded studies published before 2006, the study period ranged from 1996 to 2009. The details of the included studies were presented in Table 2, including the definitions and classifications of hospital volume, outcomes, sample characteristics, and risk adjustments. Each study was characterized by an observational design, and 5 of the 14 studies used administrative data. All studies had a sample size of >1000 patients and were population based. The numbers of patients, hospitals, and the definitions of high-volume groups and low-volume groups varied widely among the included studies. The parameters used for risk adjustments also differed substantially. A quality assessment of the included studies is presented in Figure 2.

**TABLE 1:**
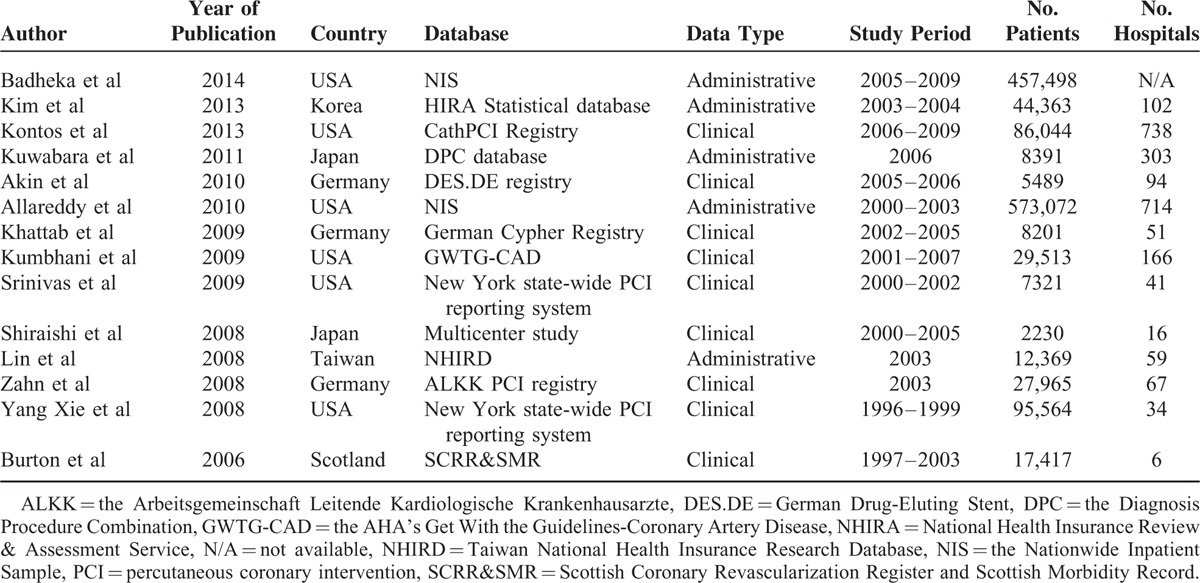
Characteristics of the Included Studies

**TABLE 2:**
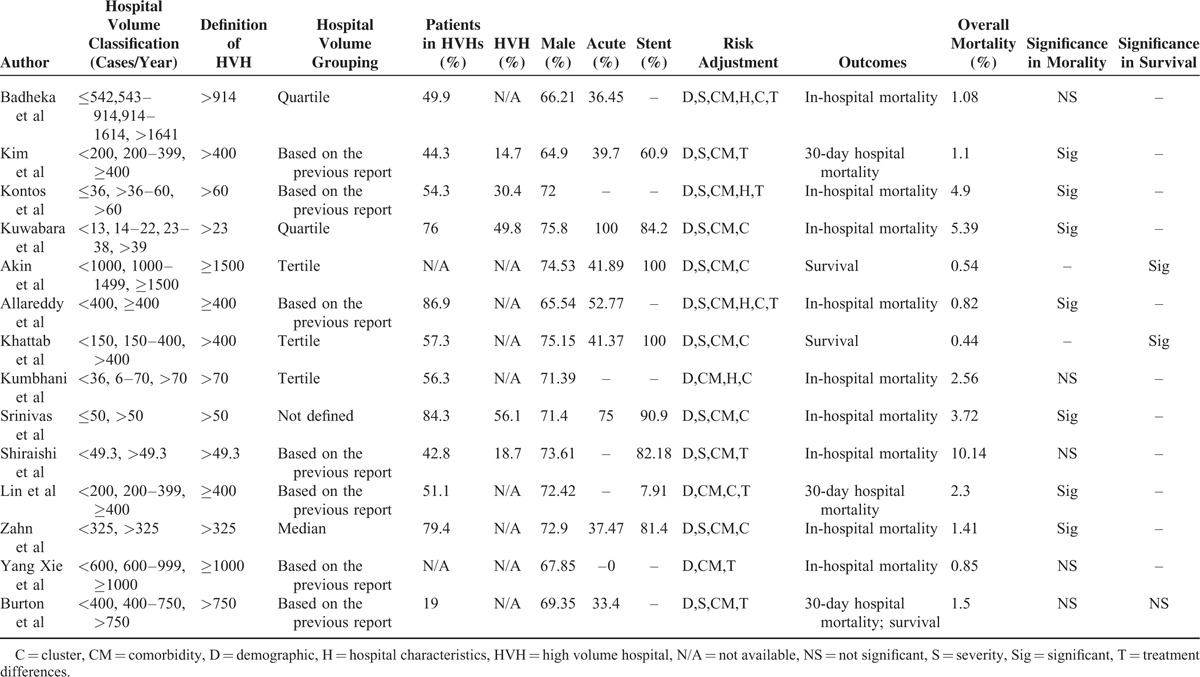
Hospital Volume Data, and Outcomes of the Included Studies

### Hospital Volume and Postoperative Mortality

Twelve studies^1,2,18–27^ that evaluated the relationship between hospital volume and postoperative mortality following PCI were included in the meta-analysis. In 7 of these studies, a significant inverse relationship between hospital volume and either 30-day or in-hospital mortality was observed.

**FIGURE 3:**
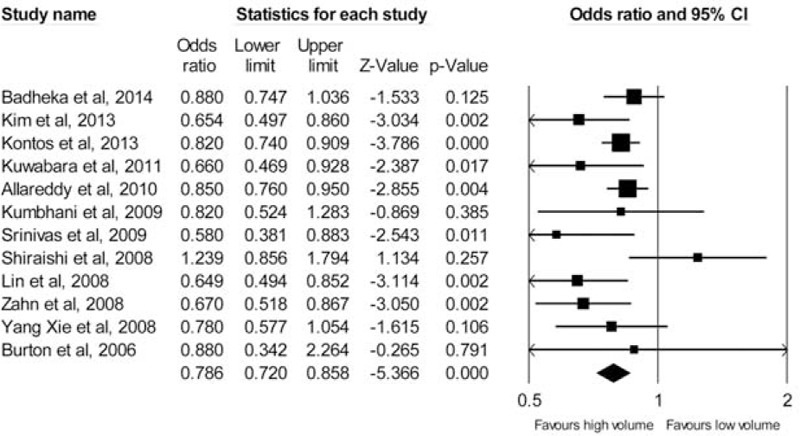
Results of meta-analysis of studies evaluating the effect of hospital volume on postoperative mortality after percutaneous coronary intervention.

**FIGURE 4:**
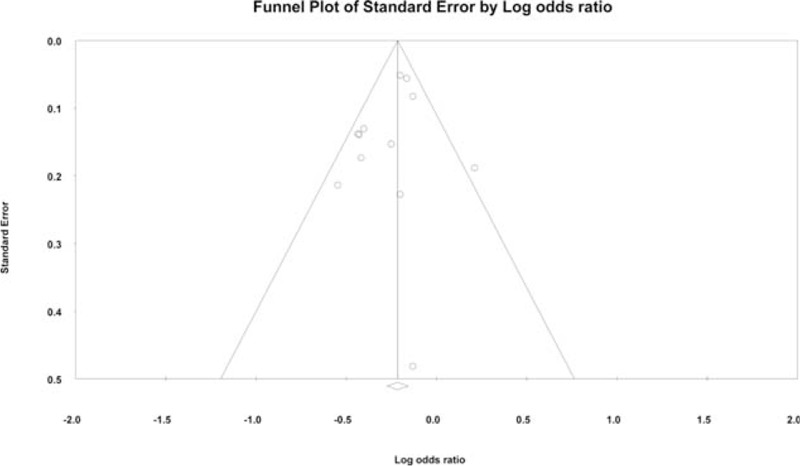
Funnel plot of standard error by log odds ratio for studies investigating the effect of hospital volume on postoperative mortality after percutaneous coronary intervention.

Figure 3 depicts the forest plot of the studies regarding hospital volume and mortality. The pooled effect estimate was significantly in favor of the high-volume providers (OR: 0.79, 95% CI: 0.72–0.86; *P* < 0.01). The analysis of the pooled effect sizes was moderately heterogeneous (*I*^2^ = 37.8%, *P* = 0.09). The funnel plot of the standard error by log OR was not suggestive of publication bias (*P* = 0.29) (Figure 4).

### Sensitivity Analysis

To check the robustness of the above pooled effect estimate and identify potential outliers, we performed a sensitivity analysis for mortality by individually removing each study included in this meta-analysis (see Table 2, Supplemental Content, which presents the results of sensitivity analyses). Omitting Badheka et al^2^ (the definition of highest hospital volume of this study is immensely different when compared with other studies) from the analysis slightly increased the heterogeneity (*I*^2^ = 39%, *P* = 0.088) but exerted only a marginal effect on the overall effect estimate (OR: 0.77, 95% CI: 0.70–0.85; *P* < 0.01). Omitting Shiraishi et al^24^ (the only study demonstrating the adverse effects of high volume) reduced the heterogeneity (*I*^2^ = 19%, *P* = 0.26), but barely changed the overall effect estimate (OR: 0.78, 95% CI: 0.73–0.84; *P* < 0.01). Removal of 4 studies from Asia^19,22–24^ resulted in a higher OR and a lower heterogeneity. The pooled OR of the 8 remaining studies was 0.82 (95% CI: 0.77–0.87, *P* < 0.01), which was similar to the result when including 12 studies, favoring the high-volume group significantly without heterogeneity (*I*^2^ = 0%, *P* = 0.51). Overall, the results of pooled effect estimate were considered robust.

### Subgroup Analysis

Following factors were included in the subgroup analysis: study country, year of publication, sample size, degree of centralization (according to the proportion of patients in HVH), definition of HVH, overall mortality, data type, and case-mix adjustment. Studies without adjustment for hospital characteristics (OR: 0.68, 95% CI: 0.57–0.80; *I*^2^ = 0.00) were associated with a larger decrease in postoperative mortality in high-volume hospitals than studies with adjustments for hospital characteristics (OR: 0.83, 95% CI: 0.78–0.88; *I*^2^ = 38.67). Furthermore, studies defining 30-day mortality seemed to report a slightly smaller effect size than studies defining in-hospital mortality (*P* = 0.05). The remaining subgroup analyses showed no statistical significance (Table 3).

**TABLE 3:**
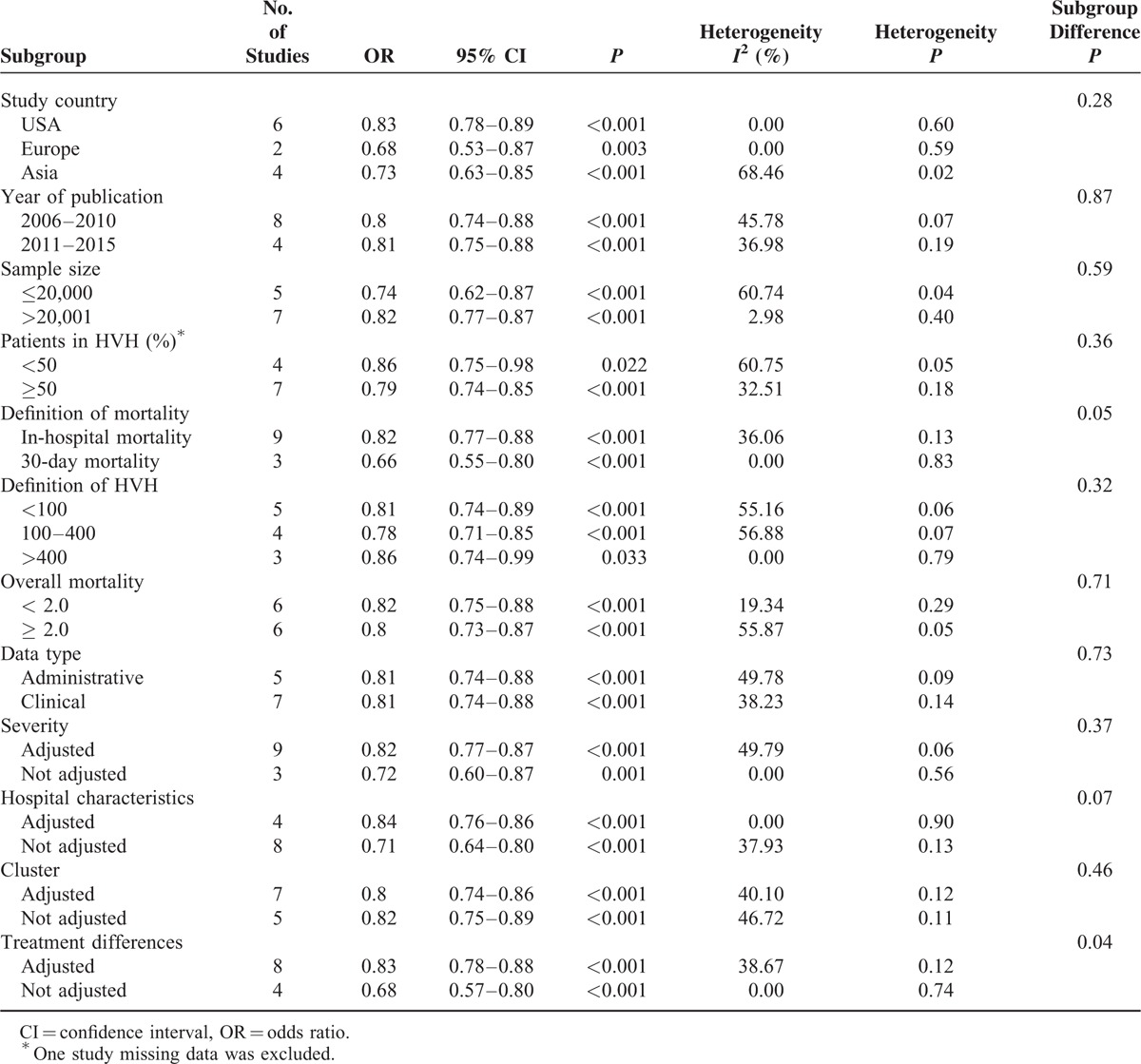
Subgroup Analyses for Mortality Outcome

### Meta Regression

The meta-regression failed to identify a relationship between the proportion of patients with acute lesions undergoing PCI and the strength of the volume–outcome relationship (*P* = 0.29), nor were any relationships identified regarding the proportion of patients requiring stents (*P* = 0.48), the proportion of male patients (*P* = 0.31), the publication year (*P* = 0.41), the overall mortality (*P* = 0.38), and the cut-off points used (*P* = 0.41 for the upper cut-off point; *P* = 0.45 for the lower cut-off point) (see Figures 1–6, Supplemental Content, which demonstrates the results of meta-regression).

### Hospital Volume and Survival

The differences in survival between high-volume and low-volume hospitals were evaluated in 3 studies published after 2006;^18,28,29^ however, only 1 study reported HRs,^18^ and the results of those studies were extremely heterogeneous. Taking account of the difficulty in assessing the magnitude of the volume effect on survival patients, we only conducted a systematic review.

Burton et al^18^ analyzed the administrative data of 17471 PCIs from 1997 to 2003, and a survival benefit was observed in high-volume hospitals with follow-up periods of 2 years, although this result was also not significant (HR: 0.85, 95% CI: 0.57–1.26). Although 2 additional studies^28,29^ did not report HRs regarding the relationship between hospital volume and survival, the Kaplan–Meier curves in the results sections of these articles demonstrated a significant effect in favor of high-volume hospitals at both 6 months and 1 year of follow-up. In summary, a trend toward better survival in high-volume hospitals was observed, although the strength of this trend was not determined.

## DISCUSSION

Our systematic review and meta-analysis examined the effect of hospital volume on the outcomes of PCI and noted a strong inverse relationship between high-volume providers and postoperative mortality (both in-hospital and 30-day mortality); however, there was moderate heterogeneity among the studies included in the analysis. To the best of our knowledge, our study is the first systematic review regarding the relationship between hospital volume and long-term survival following PCI. A trend toward higher survival in high-volume settings was observed. A meta-analysis of the relationship between hospital volume and mortality was performed previously,^12^ but said analysis did not include articles published after 2008. We reviewed the literature published until May, 2015, and corrected a data extraction error in the study by Shiraishi et al^24^ which may have affected the pooled effect estimate in previous review. Ten new studies were included in our meta-analysis, with studies originating from Japan, Germany, Scotland, and Taiwan, which improved the generalizability of our results. Moreover, we included only studies reporting ORs in our meta-analysis instead of calculating rate ratios and CIs via adjusted rates, which limited selection bias. In comparison with previous review, the present study revealed an important finding that the differences in study country affected overall heterogeneity. A possible explanation is that the quality of medical resource, financial support on hospitals, capability of training of physicians and supporting staff, and level of health information system, which may affect heterogeneity, vary considerably across different countries. In our meta-analysis, we obtained the pooled effect estimates for postoperative mortality without heterogeneity after excluding the studies from Asia, but did not significantly change the effect size. Although we exclude studies published before 2006, the study period ranges from 1996 to 2009, which could reflect the PCI practice and perioperative management in modern era. The pooled estimates for postoperative mortality favored the high-volume hospital group, which is consist with the previous review.^12^ This finding indicates that changes in PCI practices and perioperative management in recent years did not affect the persistence of the hospital volume–outcome relationship.

Moderate heterogeneity was observed among the 12 studies included in our analysis, which was not surprising given the differences in patient characteristics, study periods, and adjustments for confounding factors. To reduce heterogeneity, we adopted stricter inclusion criteria with respect to study publication year. To analyze heterogeneity, we conducted a detailed subgroup analysis; the meta-regression analysis yielded no evidence supporting the hypothesis that the strength of this relationship is associated with the proportion of patients with acute lesions or the proportion of patients receiving stents. Despite the heterogeneity observed in the analysis, this study has provided strong evidence of better short-term outcomes when PCI is conducted at high-volume hospitals. Our study supports the recommendations stipulating that institutions should achieve a higher annual PCI volume via either regionalization or consolidation, but the recommended minimal volume standard was not addressed by this paper because we compared only the highest volume group with the lowest volume group when various cut-off points were used for the studies included in our analysis.

In the future, the relationship between hospital volume and long-term outcome following PCI is worth exploring since most PCIs were performed for patients with chronic disease such as hypertension, diabetes, and chronic heart failure. Only 3 of the included studies demonstrated the results of long-term survival.^18,28,29^ We attempted to conduct a meta-analysis to analyze the long-term survival, but the poor data quality fails to support our work. To date, the relationship between hospital volume and long-term survival outcome among the patients with PCI remains unclear, but a trend toward better survival in high-volume hospitals is observed in the present study. Further research is required to confirm this trend.

An important question to address is the underlying mechanism affecting the volume–outcome relationship. Only limited numbers of studies have explored the mechanism driving the volume–outcome relationship with respect to PCI. Navarese et al^30^ analyzed the impact of time-to-presentation in the volume–outcome relationship, and the greatest benefit was observed in high-risk patients presenting within 90 minutes. Gonzalez et al^31^ evaluated the role played by failure of rescue in the volume–outcome relationship and noted a disparity of rescue ability between high-volume and low-volume hospitals. At the beginning of this century, Halm et al^32^ proposed a conceptual model demonstrating how volume is related to health outcomes and noted that factors such as physician skill and the availability of certain resources play a role in patient care. Based on this conceptual framework, Mesman et al^33^ identified the following 3 primary categories of factors: compliance with evidence-based processes of care, level of specialization, and hospital-level factors; unfortunately they did not identify conclusive set of factors. The actual mechanisms underlying the volume–outcome relationship are elusive and complex, and many aspects of hospital operations are worth pondering and exploring. More research is warranted to clarify the underlying mechanisms of the relationship between volume and outcomes.

The relationship between hospital volume and patient outcomes has important implications for patient choices, quality improvement, and the regionalization of PCI. From a patient's perspective, volume information may serve as intuitive and convenient reference to judge expected outcomes when detailed quality information is unavailable. In the perspective of quality improvement, volume, which acts as a proxy variable of outcomes, could not bring better outcomes directly, thus identifying the best practices in quality improvement is more important, including the introduction of innovative treatments and technologies, establishing multidisciplinary medical teams, the conduction of training programs, the provision of optimal care, and the guarantee of continuity of patient care. From a policy maker perspective, the translation of studies’ results into policy is difficult and complex. On one hand, the centralization of PCI would actuate a batch of high-volume providers; however, more low-volume providers would appear simultaneously because only high-volume providers would perform procedures. The problem like how low-volume providers would improve their ability to perform PCI may emerge. On the other hand, patient choice, disease incidence, healthcare resources, and medical technologies are dynamic rather than static; centralization models may not account for frequent changes in the above factors. Therefore, centralization should be closely combined with local resources and patient needs.

There were several limitations to this study. The first concern is the heterogeneity observed among included studies. Although we restricted the inclusion criteria, large variation was observed in the sample size, the definition of HVH, methods for risk adjustment, and the overall morality. However, we analyzed the heterogeneity carefully by conducting subgroup analysis and meta-regression. We found that the adjustment for treatment differences is a potential explanation of heterogeneity. Different definitions of mortality, adjustment for hospital characteristics, and potential overlap of patients may explain some of the unexplained heterogeneity.

Additionally, potential overlap of patients exists in 4 studies from USA.^2,20,21,26^ As determining the extent of overlap quantitatively was difficult for us, we decided to conduct sensitivity analyses to exclude those overlapping studies. The results of sensitivity analyses showed that excluding those overlapping studies did not alter our conclusion, mitigating the concern about overlapping.

## CONCLUSIONS

In conclusion, the present systematic review and meta-analysis revealed that postoperative mortality following PCI correlates significantly and inversely with hospital volume. A trend toward higher survival in high-volume settings has been noted; however, the effect of volume on survival is difficult to assess. To clarify the volume–outcome relationship following PCI, additional research with rigorous methodological case mix adjustment is necessary to confirm our findings and to elucidate the mechanism.

## Supplementary Material

Supplemental Digital Content
