# Supplementary material for: A Systematic Review and Meta-Analysis of the Relationship Between Hospital Volume and the Outcomes of Percutaneous Coronary Intervention
Source: Medicine (Baltimore). 2016 Feb 8;95(5):e2687. doi: 10.1097/MD.0000000000002687 (PMC4748925; doi:10.1097/MD.0000000000002687)
Supplement: Supplemental Digital Content [file medi-95-e2687-s001.doc]

**Supplemental Digital Content**

**Article title:** A Systematic Review and Meta-analysis of the Relationship between Hospital Volume and the Outcomes of Percutaneous Coronary Intervention

**First author:** Xiaojun Lin

**Figure 1.** Results of meta-regression of proportion of overall mortality of patients treated with percutaneous coronary interventions by effect size (the more negative log odds ratio, the stronger the effect size).

**Supplemental Digital Content**

**Article title:** A Systematic Review and Meta-analysis of the Relationship between Hospital Volume and the Outcomes of Percutaneous Coronary Intervention

**First author:** Xiaojun Lin

**Figure 2.** Results of meta-regression of proportion of male patients treated with percutaneous coronary interventions by effect size (the more negative log odds ratio, the stronger the effect size).

**Supplemental Digital Content**

**Article title:** A Systematic Review and Meta-analysis of the Relationship between Hospital Volume and the Outcomes of Percutaneous Coronary Intervention

**First author:** Xiaojun Lin

**Figure 3.** Results of meta-regression of proportion of acute lesion of patients treated with percutaneous coronary interventions by effect size (the more negative log odds ratio, the stronger the effect size).

**Supplemental Digital Content**

**Article title:** A Systematic Review and Meta-analysis of the Relationship between Hospital Volume and the Outcomes of Percutaneous Coronary Intervention

**First author:** Xiaojun Lin

**Figure 4.** Results of meta-regression of proportion of stent of patients treated with percutaneous coronary interventions by effect size (the more negative log odds ratio, the stronger the effect size).

**Supplemental Digital Content**

**Article title:** A Systematic Review and Meta-analysis of the Relationship between Hospital Volume and the Outcomes of Percutaneous Coronary Intervention

**First author:** Xiaojun Lin

**Figure 5.** Results of meta-regression of publication year of patients treated with percutaneous coronary interventions by effect size (the more negative log odds ratio, the stronger the effect size).

**Supplemental Digital Content**

**Article title:** A Systematic Review and Meta-analysis of the Relationship between Hospital Volume and the Outcomes of Percutaneous Coronary Intervention

**First author:** Xiaojun Lin

**
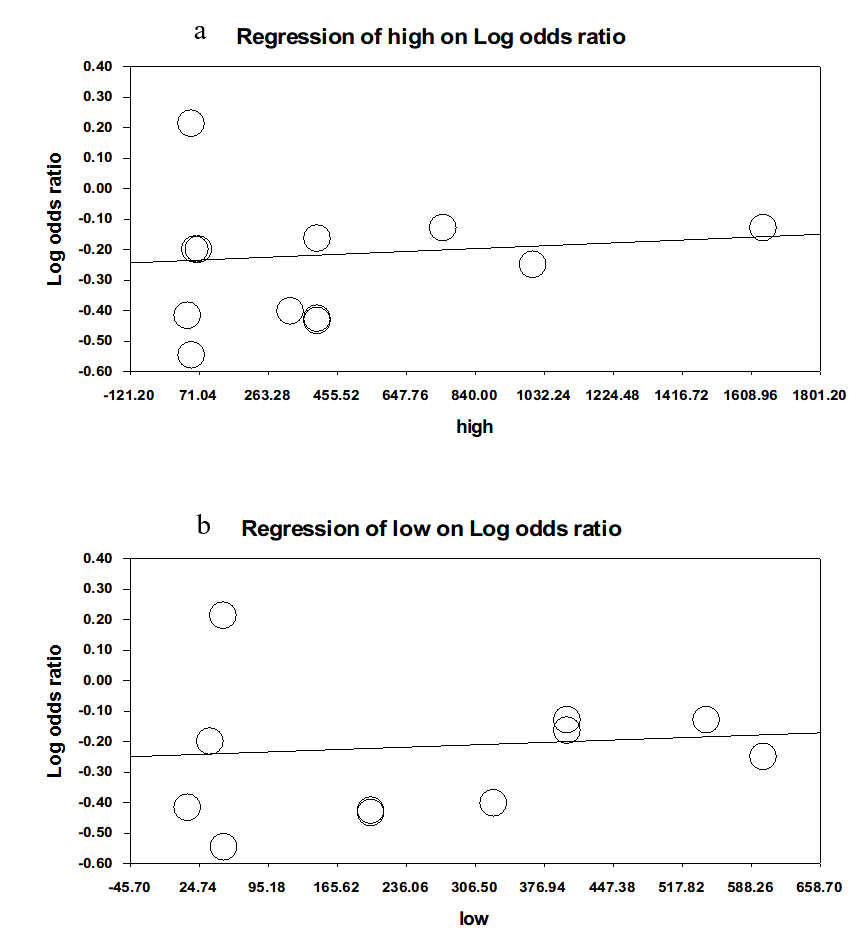
Figure 6.** Results of meta-regression of (a) high cut-off points for hospital volume and (b) low cut-off points for hospital volume (the more negative log odds ratio, the stronger the effect size).

**Supplemental Digital Content**

**Article title:** A Systematic Review and Meta-analysis of the Relationship between Hospital Volume and the Outcomes of Percutaneous Coronary Intervention

**First author:** Xiaojun Lin

**Table 1.** Search Strategies used in the databases PubMed, Embse and the Cochrane Library

| Step |  | PubMed | Embase | Cochrane |
| --- | --- | --- | --- | --- |
| 1 | (Percutaneous Coronary Intervention*[Title/Abstract]) OR (Percutaneous Coronary Revascularization*[Title/Abstract]) OR (Coronary Balloon Angioplast*[Title/Abstract]) OR (Transluminal Coronary Balloon Dilation[Title/Abstract]) OR (Angioplast*[Title/Abstract]) OR (Endoluminal Repair*[Title/Abstract]) OR (Percutaneous Transluminal Angioplasty[Title/Abstract]) | 56042 | 79309 | 8711 |
| 2 | “Percutaneous Coronary Intervention”[Mesh:noexp] OR Angioplasty[Mesh:noexp] | 10200 | 106896 | 4974 |
| 3 | Step 1 OR step 2 | 60614 | 119840 | 8738 |
| 4 | High-Volume Hospitals[MeSH] | 326 | 414 | 7 |
| 5 | Workload[MeSH] | 16110 | 30466 | 326 |
| 6 | (((“hospital volume*”[Title/Abstract] OR “admission volume*”[Title/Abstract] OR “procedural volume*”[Title/Abstract] OR “procedure volume*”[Title/Abstract] OR “Provider volume*”[Title/Abstract] OR “Institutional volume*”[Title/Abstract] OR “surgeon volume*”[Title/Abstract] OR “Operator volume*”[Title/Abstract] OR “Operative volume*”[Title/Abstract] OR “case volume*”[Title/Abstract] OR “operation rate*”[Title/Abstract] OR “surgical volume*”[Title/Abstract] OR “Workload*”[Title/Abstract] OR Caseload*[Title/Abstract] OR “high volume*”[Title/Abstract] OR “high-volume*”[Title/Abstract] OR “higher volume*”[Title/Abstract] OR “low volume*”[Title/Abstract] OR “lower volume*”[Title/Abstract] OR “low-volume*”[Title/Abstract] OR “lower-volume*”[Title/Abstract] OR “highest-volume*”[Title/Abstract] OR “lowest-volume*”[Title/Abstract] OR regionalization*[Title/Abstract] OR regionalisation*[Title/Abstract] OR “health facility size”[Title/Abstract]))) OR ((case[Title/Abstract] AND load[Title/Abstract]) OR (work[Title/Abstract] AND load[Title/Abstract])) | 52861 | 66560 | 5245 |
| 7 | Step 4 OR step 5 OR step 6 | 64239 | 82262 | 5245 |
| 8 | Mortality [Mesh] OR Morbidity [Mesh] OR Survival Rate [Mesh] OR Survival [Mesh] OR Disease-Free Survival [Mesh] OR Postoperative Complications [Mesh] OR Treatment Outcome [Mesh] OR treatment outcome[Mesh] OR Outcome and Process Assessment (Health Care)[Mesh] OR Quality of Life[Mesh] | 124712 | 2561612 | 148467 |
| 9 | (mortalit*[Title/Abstract] OR morbidit*[Title/Abstract] OR “Survival Rate*”[Title/Abstract] OR Survival[Title/Abstract] OR complication*[Title/Abstract] OR “treatment outcome”[Title/Abstract] OR “Volume– outcome”[Title/Abstract] OR outcom*[Title/Abstract] OR “Outcome and Process Assessment”[Title/Abstract] OR “Quality of life”[Title/Abstract]) | 2473269 | 3297952 | 294457 |
| 10 | Step 8 OR step 9 | 2507030 | 4243741 | 308767 |
| 11 | Step 3 AND step 7 AND step 10 | 407 | 514 | 53 |

**Supplemental Digital Content**

**Article title:** A Systematic Review and Meta-analysis of the Relationship between Hospital Volume and the Outcomes of Percutaneous Coronary Intervention

**First author:** Xiaojun Lin

| **Table 2.** Sensitivity analyses for mortality by individually removing each study | | | | |
| --- | --- | --- | --- | --- |
| **Removed study** | **OR** | **95% CI** | **Heterogeneity *I^2^*(%)** | **Heterogeneity *P*** |
| Badheka, et al | 0.77 | 0.70-0.85 | 39.07 | 0.088 |
| Kim, et al | 0.80 | 0.73-0.87 | 34.80 | 0.120 |
| Kontos, et al | 0.77 | 0.69-0.86 | 42.93 | 0.064 |
| Kuwabara, et al | 0.79 | 0.73-0.87 | 38.72 | 0.091 |
| Allareddy, et al | 0.77 | 0.69-0.85 | 39.35 | 0.086 |
| Kumbhani, et al | 0.78 | 0.71-0.86 | 43.41 | 0.061 |
| Srinivas, et al | 0.80 | 0.73-0.87 | 34.51 | 0.123 |
| Shiraishi, et al | 0.78 | 0.73-0.84 | 19.17 | 0.261 |
| Lin, et al | 0.80 | 0.73-0.87 | 33.86 | 0.128 |
| Zahn, et al | 0.80 | 0.73-0.87 | 35.83 | 0.112 |
| Yang Xie, et al | 0.78 | 0.71-0.86 | 43.28 | 0.062 |
| Burton, et al | 0.78 | 0.72-0.86 | 43.33 | 0.061 |
| OR, odds ratio; CI, confidence interval. | | | | |
